# Wireless Non‐Invasive Monitoring of Cholesterol Using a Smart Contact Lens

**DOI:** 10.1002/advs.202203597

**Published:** 2022-08-17

**Authors:** Hayoung Song, Haein Shin, Hunkyu Seo, Wonjung Park, Byung Jun Joo, Jeongho Kim, Jeonghyun Kim, Hong Kyun Kim, Jayoung Kim, Jang‐Ung Park

**Affiliations:** ^1^ Department of Materials Science and Engineering Center for Nanomedicine Institute for Basic Science (IBS) Yonsei University Seoul 03722 Republic of Korea; ^2^ Department of Biomedical Science The Graduate School Kyungpook National University 680 Gukchebosang‐ro, Jung‐gu Daegu 41944 Republic of Korea; ^3^ Department of Electronics Convergence Engineering Kwangwoon University Seoul 01897 Republic of Korea; ^4^ Department of Ophthalmology Bio‐Medical Institute School of Medicine Kyungpook National University Hospital 130 Dongdeok‐ro, Jung‐gu Daegu 41944 Republic of Korea; ^5^ Department of Medical Engineering College of Medicine Yonsei University Seoul 03722 Republic of Korea; ^6^ KIURI Institute Yonsei University Seoul 03722 Republic of Korea

**Keywords:** cholesterol, healthcare device, smart contact lens, stretchable antenna, wireless communication

## Abstract

Herein, a wireless and soft smart contact lens that enables real‐time quantitative recording of cholesterol in tear fluids for the monitoring of patients with hyperlipidemia using a smartphone is reported. This contact lens incorporates an electrochemical biosensor for the continuous detection of cholesterol concentrations, stretchable antenna, and integrated circuits for wireless communication, which makes a smartphone the only device required to operate this lens remotely without obstructing the wearer's vision. The hyperlipidemia rabbit model is utilized to confirm the correlation between cholesterol levels in tear fluid and blood and to confirm the feasibility of this smart contact lens for diagnostic application of cholesterol‐related diseases. Further in vivo tests with human subjects demonstrated its good biocompatibility, wearability, and reliability as a non‐invasive healthcare device.

## Graphical abstract

The authors present the soft smart contact lens that can detect cholesterol levels in tear fluids. This smart contact lens consists of an electrochemical cholesterol biosensor, an antenna, and a near‐field communication chip. This device shows stable cholesterol detection and portability. In addition, the cholesterol correlation between tear and blood is confirmed using rabbit models and human studies.

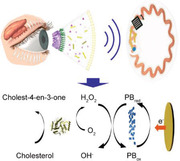

## 1 Introduction

Cardiovascular diseases (CVDs) are the leading cause of premature deaths globally, and, according to the World Health Organization (WHO), they accounted for 38% of the deaths. in 2019.^[^ ^1^ ^]^ Hyperlipidemia, a strong risk factor of CVDs, generally refers to elevations of cholesterol concentrations caused by genetic or environmental factors, including unhealthy diets.^[^ ^2^ ^]^ The cholesterol concentrations are maintained consistently by the homeostasis of cholesterol essential for the metabolic functions of the human body.^[^ ^3^ ^]^ However, excessive cholesterol due to the breakdown of the homeostasis can cause apoptosis resulting in necrotic cores which form the plaques.^[^ ^4^ ^]^ The limited blood flow by the accumulation of plaques in an artery can induce CVDs. Hyperlipidemia can be diagnosed when the total cholesterol concentration of blood exceeds the threshold of 240 mg dL^–1^ (6.2 mm). For diagnosing hyperlipidemia, the standard methods for blood cholesterol quantification are the isotope‐dilution mass spectrometry and the modified Abell–Kendall method, which are conducted in hospitals and laboratories. Although cholesterol tests are recommended every 4–6 years for healthy individuals, individuals aged 35 and older with increased risk for CVDs should have cholesterol tests more frequently, as hyperlipidemia shows no symptoms and cholesterol levels can be affected gradually by life patterns or daily diet.

Wearable healthcare devices that can monitor various physiological signals of the human body have been developed vigorously due to the increase in the awareness of wellness, which refers to the state of being in good health, and due to the advances in deformable electronics and nanotechnologies.^[^ ^5^, ^6^, ^7^, ^8^, ^9^, ^10^, ^11^, ^12^ ^]^ The 2019 pandemic of coronavirus disease caused many adverse consequences, including the restriction of movement and the necessity of individual healthcare devices for remote medical examination and treatment.^[^ ^13^ ^]^ Among various body fluids (e.g., saliva,^[^ ^14^ ^]^ sweat,^[^ ^15^ ^]^ and tears^[^ ^9^ ^]^) as decent candidates for the non‐invasive biomarker analysis for wearable healthcare devices, tears contain various types of biomarker molecules and are exposed externally facilitating easy sampling.^[^ ^16^, ^17^, ^18^, ^19^, ^20^, ^21^ ^]^ In addition, the compositions of tear fluids have shown positive correlations with blood contents because of the blood‐tear barrier, which proves the feasibility of tear fluids for monitoring disease biomarkers.^[^ ^16^, ^17^, ^18^, ^19^, ^20^, ^21^ ^]^ Therefore, the contact lens platform has emerged as a promising non‐invasive healthcare system in recent years due to its good wearability, portability, and consistent contact with the tear fluid.^[^ ^22^, ^23^, ^24^ ^]^ For example, to date, smart contact lenses (SCLs) have been developed mainly for the diagnosis of glaucoma, diabetes, and stress by measuring intraocular pressure, glucose, and cortisol.^[^ ^25^, ^26^, ^27^, ^28^, ^29^, ^30^, ^31^, ^32^, ^33^, ^34^ ^]^ In addition, the utilization of such SCL devices should be expanded further for diverse diagnostic applications and the detection of various biomarkers in tear fluids.

**Figure 1:**
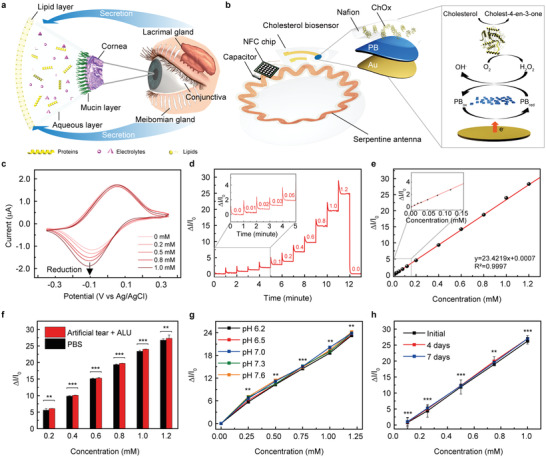
Electrochemical cholesterol biosensor in the smart contact lens. a) Schematic illustration of the tear film and secretion glands. b) Schematic illustration of the smart contact lens including a cholesterol biosensor with the enzymatic reactions. c) Cyclic voltammetry of the cholesterol biosensors for increasing concentrations of cholesterol from 0 to 1.0 mm with 0.1 mm additions in PBS solution; scan rate = 50 mV s^–1^. d) The real‐time chronoamperometric responses of the cholesterol biosensor in the range of 0 to 1.2 mm cholesterol levels in PBS solution. e) Calibration curve of the relative current change according to the cholesterol concentration (*y* = 23.4219*x* + 0.0007, *R* ^2^ = 0.9997). f) Relative changes in current with interference substances in PBS solution (red, artificial tear solution containing 10 µm ascorbic acids, 4 mm lactic acids, and 100 µm uric acid; black, PBS solution) (*n* = 10). The statistical differences between the two groups were analyzed using the unpaired student's *t*‐test. g) Calibration curves of the cholesterol biosensor according to the concentration of cholesterol with different pH ranges from 6.2 to 7.6 (*n* = 10). h) Relative changes in current immersed in PBS solution up to 7 days (*n* = 10). The error bars represent the standard deviations for (f–h). The data were analyzed using ordinary one‐way ANOVA and Bonferroni test for (g,h). Significant differences were marked as ** (*p* < 0.01) and *** (*p* < 0.001) for (f–h). Applied potential, ‐0.1 V. versus Ag/AgCl for (d–h).

Cholesterol is a modified steroid, a type of lipid. It serves as a building block for cell membranes,^[^ ^4^ ^]^ is a precursor for steroid hormones and vitamin D, and is present in tear fluid.^[^ ^25^ ^]^ Cholesterol is influenced significantly by diet and lifestyle, so continuous monitoring of cholesterol levels is essential. However, to date, the correlation of cholesterol levels between blood and tear fluid has not been studied extensively. Cholesterol exists in two forms, that is, cholesteryl ester (esterified cholesterol) and free cholesterol (unesterified cholesterol) in the tear fluid, in a 7:3 ratio.^[^ ^35^ ^]^ The tear fluid that covers the eyes (i.e., the mucin layer, aqueous layer, and lipid layer) is secreted mainly by the conjunctiva, the lacrimal gland, and the meibomian glands (**Figure** **1**a). The aqueous layer secreted from the lacrimal gland is located in the middle of the tear film.^[^ ^36^ ^]^ Tear components, secreted from the lacrimal gland and its ductal epithelium, can be associated with blood content by crossing the blood tear barrier through plasma leakage.^[^ ^37^, ^38^, ^39^ ^]^ In addition, the lipid layer is the outermost film secreted from the meibomian gland, and it consists of lipids, including wax ester and cholesteryl ester that account for the largest proportion of meibum secretion.^[^ ^40^, ^41^ ^]^ The total cholesterol in tear fluids contains a considerable portion of the cholesteryl esters secreted from the meibomian glands, while very small amounts of free cholesterol are secreted by the meibomian glands.^[^ ^40^ ^]^ Utilization of the total cholesterol level in tear fluid can be misleading when it is used to analyze the correlation of cholesterol in the blood and in tear fluid because the total cholesterol level in the blood is one of the criteria for the diagnosis of hyperlipidemia. Thus, excluding the cholesteryl ester that is the predominant substance of meibum, free cholesterol is expected to be appropriated to deduce a more reliable correlation between tear cholesterol and the disease.

Herein, we demonstrate a soft, wireless SCL for measuring the levels of free cholesterol in tear fluids using a smartphone. The integration of an electrochemical cholesterol biosensor, a stretchable wireless antenna, and an integrated circuit (IC) chip were embedded into a soft, wearable contact lens (Figure 1b). The detailed procedure for the fabrication of this cholesterol biosensor is described in Figure S1, Supporting Information, and the Experimental Section. This cholesterol biosensor was formed by incorporating cholesterol oxidase enzyme for the selective detection of the cholesterol in tears and optimized further to achieve a wide dynamic range covering tear cholesterol levels for both healthy people and patients with hyperlipidemia. To implement a wearable healthcare device, the wireless communication system was established through a stretchable antenna using a serpentine geometry, capacitors, and an IC chip for near‐field communication (NFC) enabling the use of a smartphone to transfer power and data wirelessly. The resulting SCL was validated through an in vivo study using a rabbit that was fed cholesterol by inducing hyperlipidemia. The result confirmed that feeding the rabbit cholesterol induced hyperlipidemia and the rabbit had abnormally high blood cholesterol levels. In addition, our SCL device measured in real‐time the fluctuation in the concentrations of cholesterol in the tears, revealing the correlation of cholesterol levels in the blood and tear fluid. Furthermore, the possibility of clinical applications was demonstrated through a human pilot study wearing SCLs based on the verified biocompatibility for rabbits and humans.

## 2 Results and Discussion

### 2.1 Electrochemical Cholesterol Biosensor

The electrochemical cholesterol biosensor utilizes a two‐electrode system that consists of a working electrode (WE) and a reference electrode (RE). This cholesterol sensor was designed to offer a selective recognition of cholesterol enabling continuous and repetitive measurement of free cholesterol in tear fluids through enzymatic reactions with cholesterol oxidase (ChOx). The enzyme (ChOx) was immobilized using Nafion (as a cation exchange polymer) on the WE to entrap this enzyme on the surface of the electrode and to reject interferences in the tear fluids. When the immobilized ChOx contacted free cholesterol, it catalyzed the oxidation of cholesterol to generate (cholest‐4‐en‐3‐one) and H_2_O_2_, as shown in Equation (1).

(1)

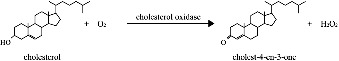




Prussian blue (PB), printed on the WE, was used as an artificial peroxidase to reduce H_2_O_2_.^[^ ^42^ ^]^ In order to translate the reaction into electrochemical response, chronoamperometry (CA) was applied by biasing −0.1 V (vs Ag/AgCl). The applied potential was selected from the cyclic voltammetry result, and the reduction potential of H_2_O_2_ was identified (Figure 1c). Upon CA operation, the reduction current of H_2_O_2_ was obtained, and it was proportional to the amount of cholesterol. Such an electrochemical detection is suitable for wearable biosensors to reduce the interference signal, which can be induced easily in the H_2_O_2_ oxidation current recording system as the tear fluid contains various oxidizable species (i.e., ascorbic acid, lactic acid, and uric acid).^[^ ^19^, ^20^, ^43^ ^]^ By applying the reduction potential of H_2_O_2_ operating CA, this cholesterol biosensor detected the free cholesterol from 0.01 to 1.2 mm (corresponding to 0.4 to 46.4 mg dL^–1^), and the resulting sensing response and calibration curve are shown in Figure 1d,e, respectively. This sensor showed high sensitivity with 1% current change per 0.043 mm (Supporting Information). The size of the WE was determined as a 0.5 mm radius in consideration of the current response at 1.2 mm cholesterol and the maximum readout current (−2.5 µA) of our NFC chip for further integration with SCL (Figure S2a,b). The wide linear detection range can cover the physiological tear cholesterol ranges of both healthy humans (≈0.4 mm) and hyperlipidemia patients.^[^ ^44^, ^45^ ^]^ The signal‐to‐noise ratio (SNR) was 4.29 at 0.01 mm (0.4 mg dL^–1^) and the limit of detection (LOD) was 9.91 µm (0.38 mg dL^–1^). The SNR and LOD values were calculated by Equation S2, Supporting Information. Figure 1f shows the comparison of cholesterol sensing performances tested in phosphate‐buffered saline (PBS, pH 7.4) and artificial tear fluids (pH 7.4) containing similar electrolyte compositions with human tears as well as physiologically relevant electro‐active interferences (ascorbic acid, 10 µm; lactic acid, 4 mm; and uric acid, 100 µm).^[^ ^19^, ^20^, ^43^ ^]^ The resulting sensing response turned out to be similar, implying that the cholesterol biosensor can operate adequately in human tears. In addition, the selectivity of the cholesterol biosensor against the cholesteryl ester was evaluated as demonstrated in Figure S3, Supporting Information. This was done because the study was targeting the measurement of only free cholesterol, not the cholesteryl ester form. The addition of 0.1 mm cholesterol stearate, a type of cholesteryl ester, showed negligible signals compared to the addition of 0.05 mm of free cholesterol. The biosensor was tested further at various pH values within the physiological pH range (pH 6.2 to 7.6) of human tear fluid, and negligible variations were observed in the sensing performance (Figure 1g).^[^ ^46^ ^]^ The operational stability of the biosensor was evaluated for one week, and this demonstrated that the sensitivity of biosensors was consistent for a week. The electrodes were kept at 4 °C in a PBS solution between the measurements (Figure 1h). Furthermore, this cholesterol sensor was immersed and operated in a PBS solution (0.6 mm of free cholesterol) for 6 h. As plotted in Figure S4, Supporting Information, the current change was negligible (relative standard deviation < 1%), which indicated the good stability of this sensor.

### 2.2 Wireless Communication Using a Smartphone

**Figure 2:**
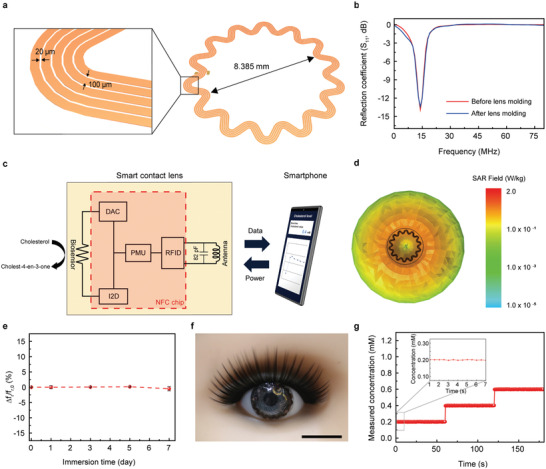
Wireless communication system of the smart contact lenses. a) Schematic illustration of the serpentine antenna. b) Resonance frequencies of the antenna before lens molding (red) and after lens molding (blue). c) Circuit diagram of the smart contact lens. d) Specific absorption rate (SAR) simulation result at 13.56 MHz. e) Relative changes in the resonance frequency of the antenna immersed in PBS solution. The error bars represent the standard deviations (*n* = 10). f) Photograph of the smart contact lens worn on the eye of a mannequin. Scale bar, 1 cm. g) Real‐time responses of the cholesterol biosensor displayed on the smartphone. Free cholesterol solution (0.2, 0.4, and 0.6 mm) was dropped on the eye of the mannequin.

Wireless communication is essential for wearable healthcare devices for convenient monitoring in human daily activities without the necessity of bulk measurement equipment.^[^ ^12^, ^14^, ^47^, ^48^, ^49^ ^]^ Thus, for the operation of SCLs, NFC was used for the wireless transmission of data and power due to its battery‐free operations and compatibility with smartphones. As mentioned earlier, the enzymatic reaction can be translated into an electrical signal output using the electrochemical amperometry method. Then, an NFC chip (NHS 3152, NXP) can collect the electrochemical signal and transmit it wirelessly to a smartphone. A serpentine structure of an antenna (inner diameter, 8.385 mm; antenna width, 100 µm; turn space, 20 µm; number of turns, 5) was used to endure mechanically stretching deformation of this soft contact lens during its molding process (**Figure** **2**a). The detailed process of the fabrication of this antenna is provided in the Experimental Section and Figure S5, Supporting Information. Moreover, good stability against mechanical deformations such as bending/stretching is a prerequisite for wearable devices.^[^ ^50^, ^51^ ^]^ Figure S6a, Supporting Information, presents relative resistance changes of the antenna attached to the surfaces of cylinders as a function of various radii from 1 to 12 mm and bending‐induced strain (*ɛ*).^[^ ^52^ ^]^ The change in resistance was negligible when the antenna was bent to a radius of curvature as small as 1 mm (*ɛ* ≤ 1.4%), which was smaller than the radius of curvature of the human eye,^[^ ^53^ ^]^ indicating that the antenna was suitable for the contact lens form. Furthermore, this antenna showed good durability in resistance under 1000 cycles of repetitive stretching (up to 30% in tensile strain at the rate of 3 mm s^–1^) (Figure S6b, Supporting Information). The antenna that was developed had an inductance of 1.05 µH, which satisfied the standardized NFC operation inductance (0.3 to 3 µH) (Supporting Information).^[^ ^54^ ^]^ In addition, an 82 pF capacitor was connected in parallel to this NFC chip (50 pF) to achieve a total capacitance of 132 pF for standardized NFC communication. The resonance frequency of this serpentine antenna with the capacitor was simulated by finite element analysis using the high frequency structure simulator (HFSS, Ansys) (Supporting Information). A hexahedral mesh structure was applied for this capacitor‐integrated antenna radiating within a cube as a medium (Figure S7a, Supporting Information). The medium was set based on the properties of air and water. The simulated resonance frequency of this antenna was 13.6 MHz in a water and air medium with a capacitance of 132 pF, as shown in Figure S7b, Supporting Information. The cholesterol biosensor, NFC chip, and capacitor were integrated further with this serpentine antenna using wire bonding interconnects. The detailed lens molding process is described in Figure S8, Supporting Information and in the Experimental Section. As shown in Figure 2b, the resonance frequency of this antenna was measured and determined to be 13.6 MHz before and after the lens molding process. The difference between the reflection coefficients (S_11_) was negligible, and the quality (Q) factor was ≈7.0 for the transmission distance of 5 mm to a smartphone. In addition, the effect of the medium on the resonance frequency of the antenna was tested further on the eye that was extracted from a rabbit (Figure S7c, Supporting Information). Although the resulting reflection coefficient was decreased when worn on a rabbit's eye, the smartphone tag still was able to translate the signal wirelessly, as the resonance frequency was consistent (Figure S7d, Supporting Information).

Figure 2c illustrates a wireless circuit diagram of the integrated SCL device including the biosensor, NFC chip, capacitor, and antenna. When a smartphone wirelessly transfers the SCL operating power, a constant voltage (−0.1 V vs Ag/AgCl) is applied to the cholesterol biosensor through a digital‐to‐analog converter (DAC) port of the chip. The generated current signal was delivered to a current‐to‐digital converter (I2D) port of this chip and then wirelessly transferred to a smartphone through the inductive coupling of the fabricated serpentine antenna (Supporting Information). The transferred signal was converted to a cholesterol concentration value (mm) using the calibrated sensing response achieved from the in vitro measurements and the resulting value was displayed on the smartphone. A specific absorption rate (SAR) was simulated with the HFSS program using the properties of the vitreous humor (permeability, permittivity, and conductivity) to evaluate the influence of electromagnetic fields on the human body as depicted in Figure 2d. The maximum SAR value of this SCL was 0.25 W kg^–1^, exceedingly lower than the international SAR standard restriction (2.0 W kg^–1^) (Supporting Information). Figure 2e shows that this SCL maintained the electromagnetic properties after it was immersed in a PBS solution for a week. The changes in the resonance frequencies were insignificant due to the parylene passivation layer (thickness, 1 µm) that encapsulated all electric components of the SCL to prevent oxidation.

As the components of the device were located at the edge of this lens, wearing SCL did not obstruct people's vision. For example, this SCL was placed on the eye of a mannequin for the real‐time measurement of various cholesterol concentrations using a smartphone (Figure 2f). Different concentrations of the solution of free cholesterol were dropped sequentially on the eye of the mannequin, and this was followed by tagging the SCL by a smartphone at a distance of 5 mm. The SCL showed reliable responses, and there was a time interval of 0.5 s between each point. The cholesterol concentration that was displayed was in good agreement with the concentration of the cholesterol that was injected, as is shown in Figure 2g.

### 2.3 Measurement of In Vivo Cholesterol in a Rabbit Model

**Figure 3:**
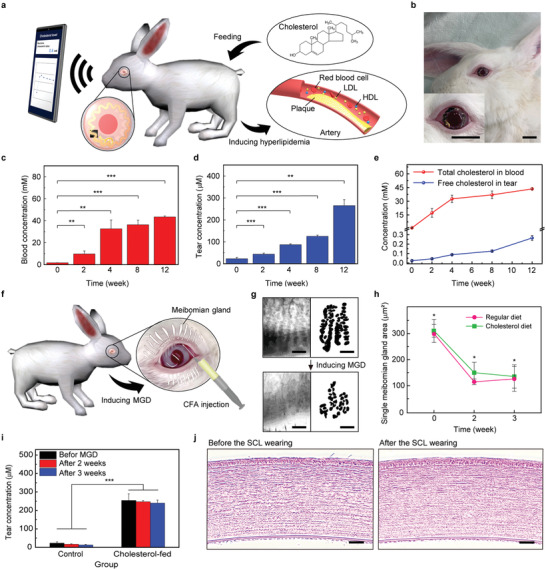
In vivo rabbit experiment. a) Schematic illustration of hyperlipidemia being induced in a rabbit by feeding the rabbit a 0.5% cholesterol chow diet. b) Photographs of a rabbit wearing the smart contact lens. Scale bars, 2 cm. c) Changes in total cholesterol concentrations of blood in the cholesterol diet‐fed rabbits (*n* = 4). d) Changes in free cholesterol concentrations of tear fluids of the cholesterol diet‐fed rabbits (*n* = 4). e) Increases in total cholesterol in the blood (red) and free cholesterol of tear fluids (blue) according to the feeding time (*n* = 4). f) Schematic illustration of inducing meibomian gland dysfunction (MGD) by the injection of Complete Freund's Adjuvant (CFA). g) Meibograhpy (left) and acini data extraction (right) in before/after (top/bottom) induing MGD. Scale bars, 0.5 mm. h) Single meibomian gland area of the control and cholesterol‐fed rabbits after inducing MGD for 3 weeks (*n* = 4). i) Changes in the cholesterol concentrations before and after inducing MGD in the control and cholesterol‐fed rabbits (*n* = 4). j) Optical microscope images of a rabbit's cornea stained with hematoxylin and eosin (H&E) of before (left) and after (right) wearing and operating the smart contact lens. Scale bars, 100 µm. The error bars represent the standard deviations for (c–e), (h,i). Significant differences were analyzed using the unpaired student's *t*‐test and marked as * (*p* < 0.05), ** (*p* < 0.01), and *** (*p* < 0.001) for (c,d,h,i).

Rabbits are used extensively as standard animal models to study atherosclerotic cardiovascular physiology (atherosclerosis), as, unlike rodent models, they have lipid metabolism that is similar to that of humans.^[^ ^55^, ^56^ ^]^ Rabbits are herbivores, and they have extremely low cholesterol concentrations (i.e., total cholesterol in the range of 20–90 mg dL^–1^, 0.52–2.33 mm), and their cholesterol levels respond rapidly and sensitively to dietary cholesterol.^[^ ^56^, ^57^ ^]^ Hence, the rabbits that were fed a diet that was high in cholesterol were expected to show rapid elevation of plasma cholesterol, which induces severe hyperlipidemia and results in the development of aortic atherosclerosis (**Figure** **3**a). Four rabbits were fed for each of the two groups, i) one with 100 g of chow diet per day that contained 0.5% cholesterol to induce hyperlipidemia and ii) another was fed with 100 g of regular chow diet each day for 12 weeks without any cholesterol consumption. In the course of feeding the rabbits cholesterol, the cholesterol contents of the blood and tears of all four rabbits were measured at 2 week or 4 week intervals. For the measurement of cholesterol in their tears, the rabbits wore SCL biosensors in an anesthetized state to reduce the unnecessary stimulation of tears by the environment or noise by movement (Figure 3b). The total cholesterol in the blood was quantified using a clinical chemistry analyzer (TBA‐120FR, Toshiba). For the rabbits that were fed a diet of cholesterol, at the starting point of this study (0 week), all four rabbits had normal total cholesterol levels in their blood, and the average value was 1.00 ± 0.29 mm. Although individual rabbits showed different speeds with which the cholesterol in their blood was elevated, all of them showed abnormally high levels of cholesterol, with an average value of 43.48 ± 0.86 mm, after 12 weeks of being fed cholesterol, as shown in Figure 3c. Figure S9, Supporting Information, shows the blood lipid profiles of the rabbits that were fed cholesterol, and the increments by which their total cholesterol levels increased were attributed to the elevation of the level of the low‐density lipoprotein‐cholesterol (LDL). This indicated that feeding the rabbits cholesterol increased their risk of having atherosclerotic cardiovascular disease. The levels of free cholesterol in tear fluids were measured by placing the smartphone close to the eye while the rabbits were wearing SCL. Then, the concentrations that were measured were processed and displayed on the smartphone screen instantaneously. Four of the rabbits showed free tear cholesterol with an average value of 22.87 ± 6.51 µm at 0 weeks and 264.9 ± 27.4 µm after 12 weeks on a 0.5% cholesterol diet (Figure 3d). Each data point was measured five times with the four rabbits and the data were averaged and error bars were shown. The cholesterol increments in the total blood cholesterol and in the free cholesterol in the tear fluid were compared in a single plot as a function of time during the 12 weeks that the rabbits were fed cholesterol (Figure 3e). Although there were individual variations, both blood and tear cholesterol concentrations were correspondingly increased in all four rabbits that were fed a cholesterol diet. For the control group with the 12‐week diet of 100 g of regular chow that contained no cholesterol, the changes in the total cholesterol concentrations in the blood and the concentrations of free cholesterol in their tear fluids are presented in Figure S10a,b, Supporting Information, respectively. The average total cholesterol concentrations in the blood of the rabbits that were fed the regular diet decreased gradually from 1.68 ± 0.17 to 0.61 ± 0.08 mm. As mentioned earlier, the range of the total cholesterol in the blood of the normal rabbits was 0.52−2.33 mm (20–90 mg dL^–1^), and all of the rabbits that were fed the regular diet showed normal cholesterol levels throughout the study. The average tear‐free cholesterol concentration for all of the rabbits in the control group was ≈28.9 ± 7.16 µm and there was no significant difference during the 12‐week period. There was ≈ninefold difference between the cholesterol of the diet‐fed rabbits (264.9 µm) and that of the control rabbits after 12 weeks of feeding. Figure S10c, Supporting Information, shows that there were negligible changes in LDL and TC, which indicated that there was no risk of any cardiovascular disorders. After 12 weeks of a cholesterol diet and a regular diet, the aortic sections of the rabbits showed the development of plaque in the blood vessels only in the group that was fed a diet of 0.5% cholesterol, confirming the progression of hypercholesterolemia^[^ ^56^ ^]^ in accordance with the results of our blood and tear cholesterol measurements (Figure S11, Supporting Information).

The mechanism of cholesterol present in tear fluids has been understudied; herein, we conducted the additional investigation with a rabbit model by inducing meibomian gland dysfunction (MGD) for the in‐depth profiling of the secretion of cholesterol by the tear glands. Unlike the lacrimal gland that generated watery tears, the meibomian tear gland secretes lipid compounds, which are known as major sources of the cholesteryl ester in tears. In addition, it may contain some portion of free cholesterol along with cholesteryl ester. Thus, MGD is expected to show an impact on the tear cholesteryl ester as well as the free cholesterol generated by the meibomian gland. MGD was induced by injecting Complete Freund's Adjuvant (CFA, F5881, Sigma–Aldrich) into the tarsal plate of an eye of the cholesterol diet‐fed rabbits and the rabbits in the control group (Figure 3f). After injecting the CFA, the obstruction of the terminal ducts and the loss of the meibomian glands were observed in all of the rabbits. As shown in Figure 3g, the meibography images of MGD‐induced regular diet‐fed rabbits showed the loss of the local portion of the meibomian glands 3 weeks after the injection. The meibomian glands were represented by the shadowed four lines (left side images) and those acini were processed further as black dots for clear presentation (right‐side images). Single meibomian gland areas for both regular diet‐fed rabbits and cholesterol diet‐fed rabbits were quantified and decreased by ≈70% (Figure 3h). Before and after the induction of MGD, both groups represented similar meibography images (Figure S12, Supporting Information). As a result, due to the obstruction and loss of the meibomian glands, the free cholesterol secretions from meibomian glands could be reduced by MGD. Figure 3i represents the gradual decrease of the free cholesterol levels for both the rabbits that were fed a normal diet and the rabbits that were fed cholesterol during 3 weeks of MGD induction, but such reduction does not have a large impact on distinguishing between rabbits with hyperlipidemia and normal rabbits. In other words, abnormally increased cholesterol for hyperlipidemia is more associated with the free cholesterol generated by the lacrimal gland rather than that generated by the meibomian gland. It also supports the possible mechanism of the presence of tear‐free cholesterol which can be similar to other small molecules in tears, showing good correlations with blood, which is based on blood plasma leakage through paracellular transport and mainly secreted by the lacrimal gland.

The safety of wearing this SCL device was evaluated regarding electromagnetic operations, and the histopathological examination was conducted with an enucleated eye of a rabbit. Figure 3j shows optical microscopies of the cornea of rabbits stained with hematoxylin and eosin (H&E), demonstrating that the electromagnetic operations did not cause the deformation of the cells. Moreover, as shown in Figure S13, Supporting Information, the immunofluorescence images exhibited no overt reaction by the cornea to the lens, and this implied that the physical damage to the cornea was negligible after wearing this SCL.

### 2.4 Demonstration With Human Participants

**Figure 4:**
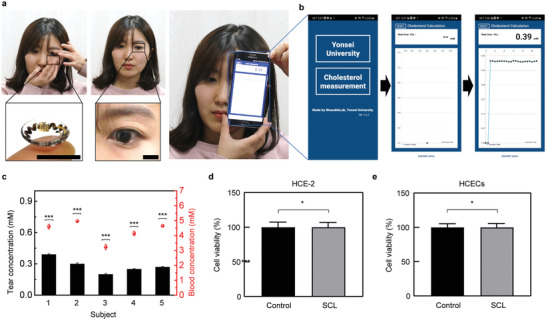
Human pilot experiment. a) Photographs of a female human subject wearing the smart contact lens and during the measurement using a smartphone. Scale bars, 2 cm. b) The sequential process of the cholesterol measurement appeared on the smartphone application. c) Cholesterol concentrations of five subjects measured using the smart contact lens (bar) and a commercial cholesterol meter (dot) (*n* = 5). d) Cell cytotoxicity test of control and the smart contact lenses with human corneal epithelial cell line (HCE‐2) (*n* = 20) and e) human conjunctival epithelial cell line (HCECs) (*n* = 20). Significant differences were analyzed using the unpaired student's *t*‐test and marked as * (*p* < 0.05) and *** (*p* < 0.001) for (c–e). The error bars represent the standard deviations for (c–e).

**Figure** **4**a; Movie S1, Supporting Information demonstrates a 25‐year‐old woman wearing this SCL and evaluating the real‐time wireless monitoring of the level of cholesterol in her tears using an external smartphone (Galaxy S6 edge, Samsung). First, the participant wore this SCL on the left eye, and, subsequently, the smartphone was placed close to the eye with a developed application turned on. The measured current response from the SCL cholesterol biosensor was transmitted wirelessly to the smartphone and then the cholesterol concentration was calculated. The resulting cholesterol concentration in the tear appeared directly on the screen of the smartphone. The sequential process displayed on the smartphone application is shown in Figure 4b. Five healthy participants volunteered, and the concentrations of cholesterol in their blood and tears were determined as depicted in Figure 4c. The aim of this process was to validate the sensing performance of SCL cholesterol in actual wearing scenarios. The free cholesterol concentrations in tear fluids were measured through the SCLs, and the total cholesterol concentrations in the blood were measured using a commercially available cholesterol meter (Barozen lipid meter, Handok) as shown in Figure S14, Supporting Information. The average concentration of free tear cholesterol from five participants turned out to be 0.28 ± 0.03 mm, similar to the normal range around 0.40 mm.^[^ ^44^, ^45^ ^]^ The normal range for the total cholesterol concentration in blood was known to be less than 5.17 mm (200 mg dL^–1^), and the average value that was measured was 4.32 ± 0.68 mm, which fit within the range, confirming that all subjects had cholesterol levels that were close to normal for both blood and tears.

Furthermore, the biocompatibility was evaluated through a cell viability test with human corneal cells (HCE‐2, #CRL‐11135, clone 50.B1, ATCC) and human conjunctival cells (HCECs, #CCL‐20‐2, clone 1–5c‐4, ATCC). When worn by humans, the SCL contacted both the cornea and the conjunctiva, and hence, cytotoxicity tests were performed on both human cells. After culturing the cells for 24 h and then adding ethidium homodimer‐1 (LIVE/DEAD Viability/Cytotoxicity kit, Invitrogen), the absorbance of each group was measured to record the survival rate of the cells. More details are provided in the Experimental Section. Figure 4d,e shows the viability of the cells of each group, and the cell viability of the SCL was 98 ± 2.07% and 99 ± 1.83%, which is defined as non‐cytotoxic according to ISO 10993–5. In addition, fluorescence microscope images of the cultivated cells stained with green fluorescent calcein‐AM are shown in Figure S15a,b, Supporting Information, which indicates no toxicity of SCL devices in either group.

## 3 Conclusion

We developed soft SCL devices that can monitor the cholesterol level in tear fluids using a smartphone wirelessly for the diagnosis and monitoring of the progression of hyperlipidemia. The electrochemical cholesterol biosensor on this contact lens platform showed selective, sensitive, and reliable sensing performance of tear cholesterol with wide dynamic ranges, allowing continuous and repetitive detections. For the wireless communication system, this sensor also was integrated with an NFC chip and a stretchable antenna on the contact lens for its wireless, battery‐free operation. The measured concentration of cholesterol in tears was displayed directly on the screen of a smartphone. The in vivo study was performed with a rabbit model, and hyperlipidemia and MGD disease were induced. This SCL demonstrated the promising correlation between the cholesterol levels in both blood and tears and the feasibility of the SCL as a diagnostic device for hyperlipidemia by measuring cholesterol levels in tear fluids. The study with MGD‐induced rabbits enabled in‐depth investigation to identify the possible mechanisms of such a positive correlation. Moreover, human trials confirmed its good wearability and reliability as a non‐invasive healthcare device along with appropriate biocompatibility. Despite the promising performance of this SCL, as the first non‐invasive healthcare device monitoring cholesterol for in vivo applications, an additional study including the evaluation of clinical data on tear cholesterol variations of individuals can be conducted to determine the potential capability of this SCL in clinical use.

## 4 Experimental Section

### Fabrication of the Cholesterol Biosensor

A 25 µm‐thick polyimide (PI) film was attached to the thoroughly rinsed SiO_2_ wafer with the aid of the spin‐coated PDMS (16 µm, 4000 rpm, 40 s) as an adhesive layer. The electrode (thicknesses of Cr/Au, 10/100 nm) was deposited by thermal evaporation on SiO_2_/PDMS/PI and the photoresist (PR, S1818, Microchem Corp.) was spin‐coated (3000 rpm, 40 s). Current collect electrodes were patterned by back ultraviolet (UV) exposure (10 mW cm^–2^, 18 s). After development (AZ 300K MIF developer, AZ electronic materials), the Cr/Au layer was wet‐etched (CE‐905N, 10 s, TFA, 40 s, Transcene). The areas of the WE and RE were 0.785 and 3.448 mm^2^, respectively. The remaining PR was exposed to UV (power: ≈10 mW cm^–2^, exposure time: 5 min, MDA‐600S, Midas System) and removed by a developer (AZ 300K MIF). After the remaining PR was removed, a 500 nm thick parylene layer was deposited by chemical vapor deposition (CVD) to encapsulate the interconnects of the biosensor. Then, PR was spin‐coated (2000 rpm, 40 s) and exposed to UV except for the interconnect parts, and this was followed by dry etching with oxygen plasma in reactive ion etching (RIE, 100 W, 200 mTorr of O_2_, 150 s) to open the WE and RE. Then, 25 µm‐thick PI films were used to fabricate the masks for screen‐printing. 1 µm thick‐Cu was deposited on a PI film that was 25 µm thick. Then, PR(S1818) was spin‐coated (2000 rpm, 40 s), and WE and RE shapes were patterned by an aligner (≈10 mW cm^–2^, 23 s). The exposure PR was developed by the developer (AZ 300K MIF, 50 s) and the Cu was wet‐etched (Type 1 Ni etchant, 40 s). To make WE and RE shapes, dry‐etch substrates by RIE (200 W, 600 mTorr of SF_6_, Ar, O_2_, 40 min).

The masks that were patterned via photolithography and RIE were placed on top of the substrate and fixed with adhesive tape. After UVO 15 min, Prussian blue carbon ink (PB, C2070424P2, Sun Chemical) was spread on WE with the screen‐printing mask and rubber stamp that only opened the WE by manual and annealed it at 110 °C. The mask was removed carefully. In addition, Ag/AgCl ink (Product #011464, ALS) was spread manually on RE with the screen‐printing mask and annealed at 110 °C.

Cholesterol oxidase (ChOx) (Product #C8649, Sigma–Aldrich) solution (500 mg dL^–1^) was prepared in a PBS solution (pH 7.4, Product #806552, Sigma‐Aldrich). In order to prevent the potential damage to ChOx during the lens molding process, 2 µL of the solution was dropped onto the WE (on the printed PB) after the lens molding process (Figure S8, Supporting Information). After 2 h of drying at a constant temperature (4 °C) and humidity (≈10%), 2.0 µL of 0.5% Nafion (Product #309389, Sigma–Aldrich) was dropped on the electrode to form an immobilization layer, followed by drying overnight at 4 °C.

### Electrochemical Characterization of the Cholesterol Biosensor

The 10‐mm cholesterol stock solution using PBS was prepared by adding 10 wt% triton X‐100 (Product #T8787, Sigma–Aldrich). The cholesterol biosensor was immersed in PBS, and the cholesterol stock solution was injected into PBS solution to obtain the desired concentration of cholesterol, and the incubation time was 1 min. A potentiostat (PMC‐CHS08A, Princeton Applied Research) was used to determine the electrochemical performance of the cholesterol biosensor. Cyclic voltammetry (CV) and chronoamperometry (CA) were conducted in the various concentrated cholesterol solutions. CV curves were measured in the potential window from 0.35 to −0.35 V versus Ag/AgCl (scan rate, 50 mV s^–1^). In addition, CA was measured with −0.1 V of applied potential for 1 min to record the current response from the enzymatic reaction.

### Selectivity and pH Stability Test

Considering the ion concentration of human tear fluids, the ion concentration (NaCl, 94.11 mm; KCl, 20.12 mm) of the artificial tears was achieved by mixing KCl solution in a diluted saline (Product #D8537, Sigma–Aldrich). The selectivity of the biosensor was tested by adding 10 µm ascorbic acid (Product #A92902, Sigma–Aldrich), 4 mm lactic acid (Product #PHR1113, Sigma‐Aldrich), and 100 µm urea (Product #U5128, Sigma–Aldrich) in the adjusted artificial tears, all of which are well‐known substances that exist in tear fluids. The pH of the solution was adjusted from 6.2 to 7.6 by dropwise KCl (0.1 m) additions to the artificial tears using a pH meter (Orion STAR A2116, Thermo Scientific).

### Fabrication of the Stretchable Antenna

A layer of Cr/Cu (thicknesses, 10/3000 nm) was deposited by thermal evaporation on SiO_2_/PDMS/PI. PR was spin‐coated (2000 rpm, 40 s), and the serpentine ring was patterned photolithographically. The non‐patterned Cu layer was wet‐etched by Nickel etchant (Type 1, 30 s, Transene). The PI film was dry‐etched by RIE (200 W, 600 mTorr of SF_6_, Ar, O_2_, 40 min) to form the serpentine‐shaped substrate. In addition, contact pads (thicknesses of Cr/Au, 10/100 nm) were deposited by thermal evaporation on the serpentine‐structured substrate. The antenna (thicknesses of Cr/Cu, 10/3000 nm) was deposited, followed by spin‐coating (2000 rpm, 40 s) of the PR. The serpentine antenna was patterned by an aligner (10 mW cm^–2^, 23 s), and the exposed PR was developed for 50 s. The parts where PR was developed were wet‐etched for 40 s to fabricate a serpentine antenna by Nickel etchant. A parylene layer (thickness, 1 µm) was deposited for the encapsulation of the serpentine antenna and patterned by photolithography to form holes on the inner and outer ends of the antenna. The electrical conduction path was maintained due to the Au pads (thickness, 100 nm) on both ends of the antenna.

### Wireless Electromagnetic Characterization

The electromagnetic properties of an antenna related to wireless communication were characterized using a network analyzer (Rohde & Schwarz, ZNB 8). For the immersion test to evaluate the reliable wireless communication properties of the antenna, the SCL samples were immersed in the PBS solution for a week, and the characteristics were measured using the network analyzer. In addition, to check the dielectric constant of the medium, the experiment was conducted under the same conditions even after putting SCL on the rabbit's extracted eye.

### Integration of the Wireless Cholesterol Biosensor Into a Soft Contact Lens

The cholesterol biosensor, NFC chip (NHS 3152, NXP Semiconductors), and a capacitor (82 pF, Kyocera AVX) were placed onto the antenna and connected electrically by either i) Au wires extracted from the wire bonder (7KE, Westbond) or ii) liquid–metal 3D wires printed through a glass‐capillary nozzle.^[^ ^9^, ^58^, ^59^ ^]^ The fully integrated device was embedded into a mold to form a contact lens filled with silicone elastomer (MED‐6015, Nusil). The surfaces of the WE and RE were covered by a protection film made with a black‐painted circular PI film and a PDMS thin film (50 µm). After curing the molded SCL at a pressure of 313 kPa and a temperature of 70 °C for 6 h, the SCL was embedded completely with the silicone elastomer. Then, the protective film was removed for the direct contact of the biosensor with the tear fluids.

### Rabbit Animal Study

All in vivo tests were conducted according to the guidelines of the Institutional Animal Care and Use of Committee of Daegu‐Gyeongbuk Medical Innovation Foundation (DGMIF) and the Institute of Animal Care and Use Committee of Yonsei University (DGMIF‐21070801‐01 and IACUC‐202106‐1276‐03). The Institute of Animal Care and Use Committee of DGMIF and the Institute of Animal Care and Use Committee of Yonsei University are the ethics review committee. A total of eight male New Zealand white rabbits (Samtako Korea Co., Ltd) that were 3 months old and weighed 2.0–2.5 kg were utilized for this study. After 7 days of acclimatization, the rabbits were divided randomly into two groups based on body weight, and they started on a 15‐week diet that consisted of a regular diet (Purina) and a 0.5% cholesterol diet (JA Bio, Inc.). After the diet began, at 2, 4, 8, and 12 weeks, 3 mL of blood was collected through the marginal ear vein, and the concentration of plasma cholesterol was analyzed. At the same time, the concentration of tear cholesterol was measured using the SCL. The slit‐lamp examination was conducted with the rabbits before and after wearing the SCL. A droplet (volume, 2 µL) of 2% fluorescent dye was applied to the lower conjunctival sac to evaluate the ocular surface with a slip lamp (SL‐15, Kowa Optimed). For these experiments, the rabbits were anesthetized with ketamine (35 mg kg^–1^) and xylazine (10 mg kg^–1^).

### Meibomian Gland Dysfunction (MGD)

Induction of MGD was performed after 12 weeks of experimentation, and it was observed for 3 weeks by injecting 10 µL each of CFA into the inner, middle, and outer eyelid margins of the upper right eyelid. The upper left eyelid without CFA injection was used as a control. After the CFA was injected, changes in the meibomian glands were observed and analyzed through meibography at 2 weeks and 3 weeks according to the previously‐reported meibography system.^[^ ^60^ ^]^

### Human Participant Study

The protocol for the human participant study was approved by the Institutional Review Board of Yonsei University (7001988‐202112‐HR‐1451‐02). All experiments and trials were conducted with the full, informed consent of the participants. Initially, the participants stabbed their fingertips with a needle and collected 15 µL of blood using a capillary tube in a cholesterol meter (Barozen lipid meter, i‐SENS, Inc.) to measure the cholesterol in the blood. Subsequently, the SCLs were rinsed with a PBS solution before wearing. Then, after the participants wore the SCL to their eyes, the cholesterol in their tears was measured by placing the smartphone (Galaxy 6, Samsung) close to the eyes. The participants measured the cholesterol in their blood and tears at the same time every day for 3 days, and they could stop the experiment at any time. The authors affirm that human research participants provided written informed consent for the publication of the images in Figure 4a; Figure S14 and Movie S1, Supporting Information.

### Aortic Sections of Rabbit Vessels

After the 12‐week experiment with the rabbits was completed, the rabbits were anesthetized with 5% isoflurane and then euthanized by intravenous KCl (20 mL, 10 mm). After collecting the aortic arches from the euthanized rabbits, frozen sections were performed, and intravascular plaque and lumen were observed through oil red O staining and hematoxylin and eosin staining (Figure S11, Supporting Information).

### Cell Cytotoxicity Test With Human Cell Lines

For the cytotoxicity assay, human corneal epithelial cell lines (HCE‐2, #CRL‐11135, clone 50.B1, ATCC) were cultured in DMEM/F‐12, HEPES medium (Gibco), and human conjunctival epithelial cell line (HCECs, #CCL‐20‐2, clone 1–5c‐4, ATCC) cultured in RPMI 1640 medium (Gibco). Both cell lines were used to investigate the toxicity of SCL. The SCL was sterilized with 70% ethanol for 2 min, washed three times in PBS for 2 min, and eluted in a cell culture medium as an SCL test group, containing 1% penicillin‐streptomycin (P/S, Gibco) and 2% fetal bovine serum (FBS) at 37 °C for 24 h. In addition, cell culture medium containing 1% P/S and 2% FBS was used as a control group. The cells proliferated up to about 80%, and then they were incubated at 37 °C, in 5% CO_2_ for 24 h using the negative and treated extraction fluids. Subsequently, each group was washed with sterilized PBS solution and stained with calcein‐AM (1 µL) and ethidium homodimer‐1 (4 µL) (LIVE/DEAD Viability/Cytotoxicity kit, Invitrogen) in the dark for 30 min at room temperature, followed by random images analyzed using a fluorescence microscope (BX53, Olympus). At the same time, a cell viability assay (*n* = 20) was performed. After incubation with the negative or treated extraction fluids for 24 h, the cells were treated with 10 µL of CCK‐8 reagent (Cell Counting Kit‐8, Dojindo) and incubated at 37 °C in 5% CO_2_ for 2 h. Cell viability was analyzed after measuring absorbance in a microplate reader (VersaMax, Molecular Devices) using a 450 nm filter.

### Statistical Analysis

Prism 8.0 (GraphPad, San Diego, USA) and SPSS 19.0 software (SPSS Inc., Chicago, USA) were used for statistical analysis. Data points were shown as mean ± standard deviation. The sample sizes (*n*) and statistical methods were specified in each figure legend. The statistical differences between the two groups were analyzed using the unpaired student's *t*‐test, and more than two groups were analyzed using ordinary one‐way ANOVA and Bonferroni test. Significant differences were marked as * (*p* < 0.05), ** (*p* < 0.01), and *** (*p* < 0.001).

## Conflict of Interest

The authors declare no conflict of interest.

## Author Contributions

H.S. and H.S. contributed equally to this work. *Contributed to the experiments, analyzed the data, and wrote the manuscript*: H.S. and H.S. *Participated in the rabbit experiments*: H. S. *Fabricated the contact lenses*: W. P. and B. J. J. *Participated in the in‐vivo rabbit test and cell viability test*: J. K. *Participated in the design of the wireless communication system and the analysis of electromagnetic properties*: J. K. *Contributed in the planning of the project and the animal experiments*: H. K. K. *Contributed to the design of the electrochemical biosensor and revised the manuscript*: J. K. *Oversaw all of the research phases and revised the manuscript*: J.‐U. P. All the authors discussed and commented on the manuscript.

## Supporting information

Supporting Information

Supplemental Movie 1

## Data Availability

The data that support the findings of this study are available from the corresponding author upon reasonable request.
