# Supplementary material for: Wireless Non‐Invasive Monitoring of Cholesterol Using a Smart Contact Lens
Source: Adv Sci (Weinh). 2022 Aug 17;9(28):2203597. doi: 10.1002/advs.202203597 (PMC9534953; doi:10.1002/advs.202203597)
Supplement: Supplementary file 1 — Supporting Information [file ADVS-9-2203597-s001.pdf]

Supporting Information

Wireless Non-invasive Monitoring of Cholesterol Using A Smart Contact Lens

Hayoung Song[+], Haein Shin[+], Hunkyu Seo, Wonjung Park, Byung Jun Joo, Jeongho Kim, Jeonghyun Kim, Hong Kyun Kim*, Jayoung Kim*, Jang-Ung Park**

H. Song, H Shin, H Seo, W Park, B. J. Joo, Prof. J.-U. Park
Department of Materials Science and Engineering, Center for Nanomedicine Institute for Basic Science (IBS), Yonsei University, Seoul 03722, Republic of Korea
E-mail: jang-ung@yonsei.ac.kr

J. Kim, Prof. H. K. Kim
Department of Biomedical Science, The Graduate School, Kyungpook National University, 680 Gukchebosang-ro, Jung-gu, Daegu, 41944, Republic of Korea
E-mail: okeye@knu.ac.kr

Prof. Jeonghyun Kim
Department of Electronics Convergence Engineering, Kwangwoon University, Seoul 01897, Republic of Korea
E-mail: jkim@kw.ac.kr

Prof. H. K. Kim
Department of Ophthalmology, Bio-Medical Institute, School of Medicine, Kyungpook National University Hospital, 130 Dongdeok-ro, Jung-gu, Daegu 41944, Republic of Korea
E-mail: okeye@knu.ac.kr

Prof. Jayoung Kim
Department of Medical Engineering, College of Medicine, Yonsei University, Seoul 03722, Republic of Korea
E-mail: jayoungkim@yonsei.ac.kr

Prof. J.-U. Park
KIURI Institute, Yonsei University, Seoul, 03722, Republic of Korea.
E-mail: jang-ung@yonsei.ac.kr

[+] H. Song and H. Shin contributed equally to this work.

This PDF file includes:
Supporting Methods
Supporting Figures
Captions for Supporting Movie

Supporting Methods

Sensitivity calculation of the cholesterol biosensor.

The sensitivity of the cholesterol biosensor with respect to the cholesterol concentration can be determined by Equation (1),

$$\text{Sensitivity } (S) = \frac{\Delta C}{\Delta(\frac{I-I_0}{I_0})} \quad (1)$$

where C is the concentration of free cholesterol, I is generated current of the biosensor, and I_0 is the current at 0 mM cholesterol concentration.

Limit of detection calculation of the cholesterol biosensor.

The signal-to-noise ratio (SNR) of the cholesterol biosensor can be calculated by Equation (2),

$$SNR = 20\log\frac{I_S}{I_N} \quad (2)$$

where I_S is saturated current at arbitrary cholesterol concentration, and I_N is the amplitude of noise of the cholesterol biosensor at the same cholesterol concentration. The limit of detection (LOD) of the biosensor can be determined by Equation (2) when the SNR is 3, and the I_N is the noise amplitude of the cholesterol biosensor.

Inductance and frequency simulation of antenna.

To know the antenna's target inductance, inductance was calculated by substituting frequency and the chip's capacitance to the resonant frequency Equation (3),

$$f = \frac{1}{2\pi\sqrt{LC}} \quad (3)$$

where f is frequency, L is inductance, and C is capacitance. And the calculated inductance is substituted to NFC/RFID spiral coil inductance Equation (4),

$$L = 31.33\mu_0 N^2 \frac{d}{8d+11c} \quad (4)$$

to derive the number of turns, where μ_0 is the vacuum permeability, d is the average radius, c is the width of an antenna, and N is the number of turns.

Calibration of the cholesterol readout using a smartphone.

The base current (I_0) at 0 mM of cholesterol was measured, and the second current (I_2) was measured at a specific cholesterol concentration. Then, the relative change to calibration was calculated by Equation (5).

$$\frac{\Delta I}{I_0} = \frac{I_2 - \mathrm{I}_0}{I_0} \tag{5}$$

The relative change was coded in chips to correspond to the specific cholesterol concentration. When the SCL was worn on the eye, the digital to analog converter (DAC) port of the chip applied reduction potential (vs Ag/AgCl) to the working electrode of the biosensor. And the current generated by reacting with cholesterol in tear fluid flowed into the chip through the Current to Digital converter (I2D). And this flowed current was digitized through pre-coded calibration and appeared on the smartphone's application.

Calculation of bending-induced strain

The sample thickness (t) was the total thickness of the substrate (25 µm-thick PI film) and the serpentine antenna (3 µm-thick Cr/Cu) placed on it. Also, the bending radius (r) was the radius of the cylinder with the sample attached to the surface. The bending-induced strain (ε) of the electrode at the substrate surface can be calculated by Equation (6).

$$\mathrm{Bending - induced\ strain}\ (\varepsilon) = \frac{t}{2R} \times 100\ (\%) \tag{6}$$

Simulation of the antenna resonance frequency.

The resonance frequency of the antenna was simulated using a commercial simulation program, high frequency structure simulator (HFSS, ANSYS Inc.). A hexahedral mesh

structure was applied for the antenna solid. The material properties were used as the values provided by the program (mass density, 8.933 kg m^{-3}; relative permittivity, 1; relative permeability, 0.999991; electric conductivity, 5.8×10^7 S m^{-1})

Simulation of specific absorption rate (SAR).

For the simulation of the specific absorption rate (SAR), the finite-element analysis was conducted by the commercial simulation program (HFSS, ANSYS Inc.). The hexahedral mesh structure was applied for the three-dimensional human eye model. The material properties of the eye model were adjusted as the properties of vitreous humor (relative permittivity, 68.92; relative permeability, 1; electrical conductivity, 1.61 S m^{-1}; mass density, 1.0053 kg m^{-3}).

Supporting Figures

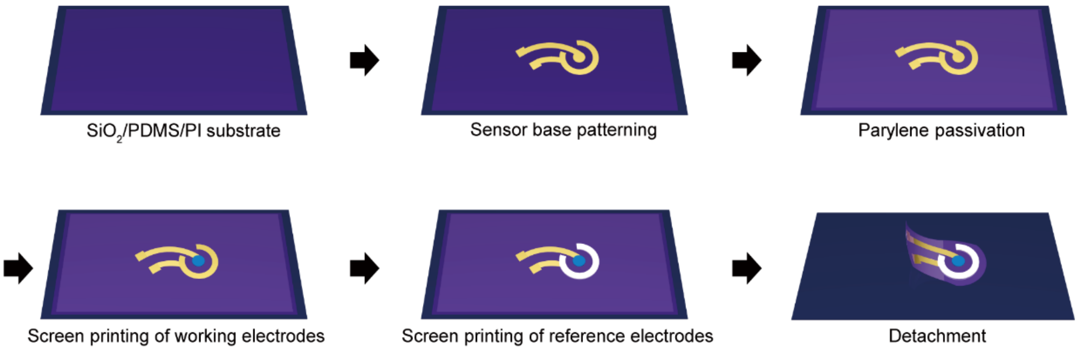

Figure S1. Schematic illustration of the fabrication process of the electrochemical cholesterol biosensor.

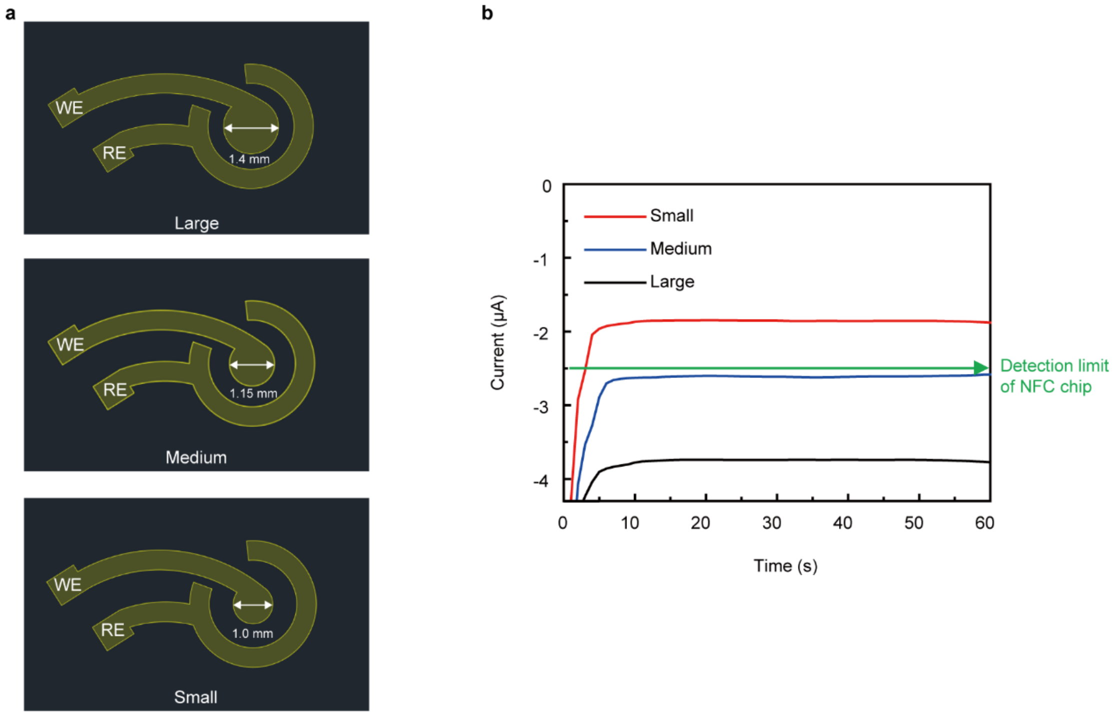

Figure S2. Size optimization of the working electrode of electrochemical cholesterol biosensors. a) Illustrations of the cholesterol biosensor with the large working electrode (top), medium working electrode (middle), and small working electrode (bottom). b) Chronoamperometric responses of the cholesterol biosensors with respective sizes of working electrodes at 1.2 mM cholesterol concentration.

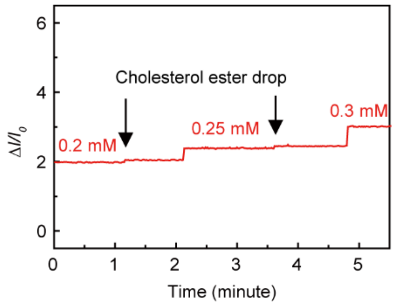

Figure S3. Selectivity of the cholesterol biosensor against cholesteryl ester. Black arrows represent the time point that 0.1 mM cholesterol stearate was dropped.

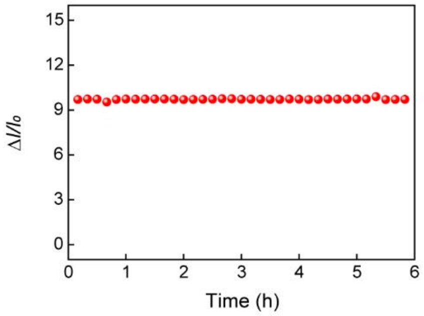

Figure S4. Long-term stability of the electrochemical response to 0.6 mM cholesterol for 6 hours operation at 10-minute intervals. Here, this cholesterol sensor was immersed and operated in a PBS solution (0.6 mM of free cholesterol) for 6 hours.

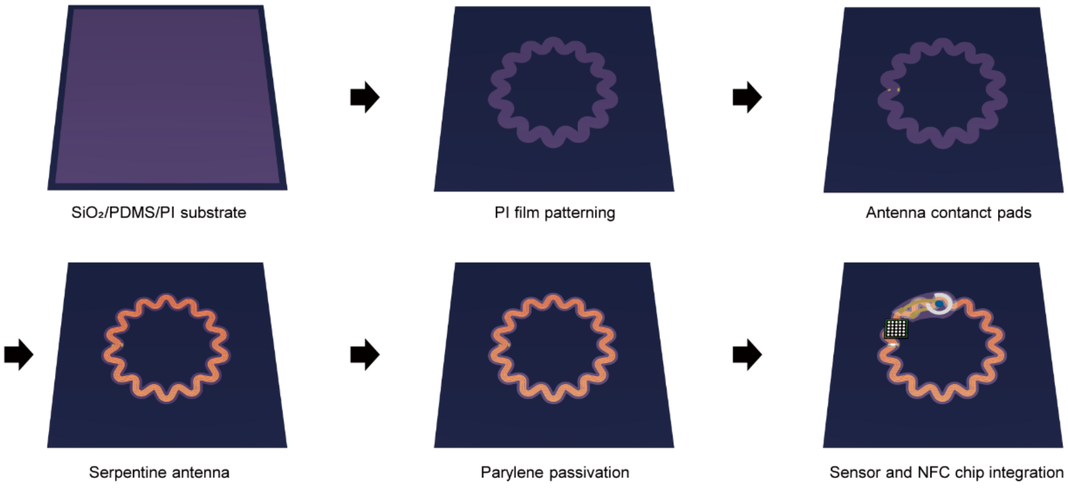

Figure S5. Schematic illustration of the fabrication process of the serpentine antenna.

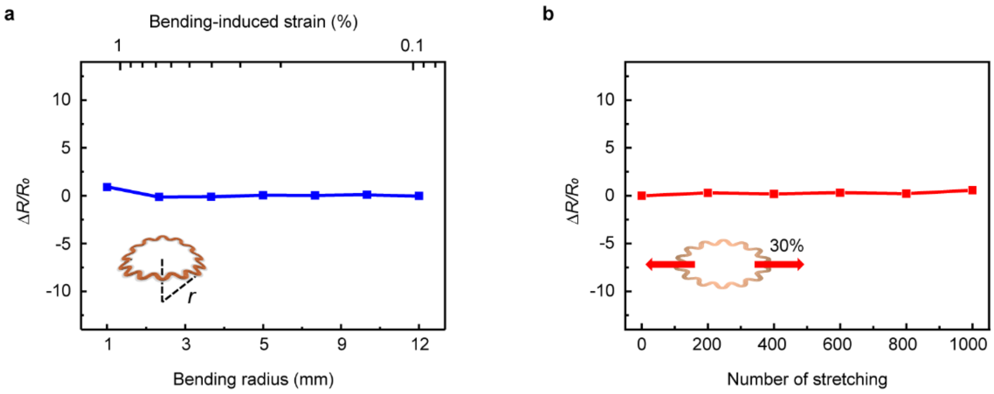

Figure S6. Mechanical stabilities of the stretchable serpentine antenna. a) Relative resistance changes as a function of the bending radius (*r*) from 1 mm to 12 mm and bending-induced strain (ε). The inset shows the bent serpentine antenna. b) Relative resistance changes of the antenna after repetitive stretching (30% of tensile strain). The inset shows the stretched serpentine antenna

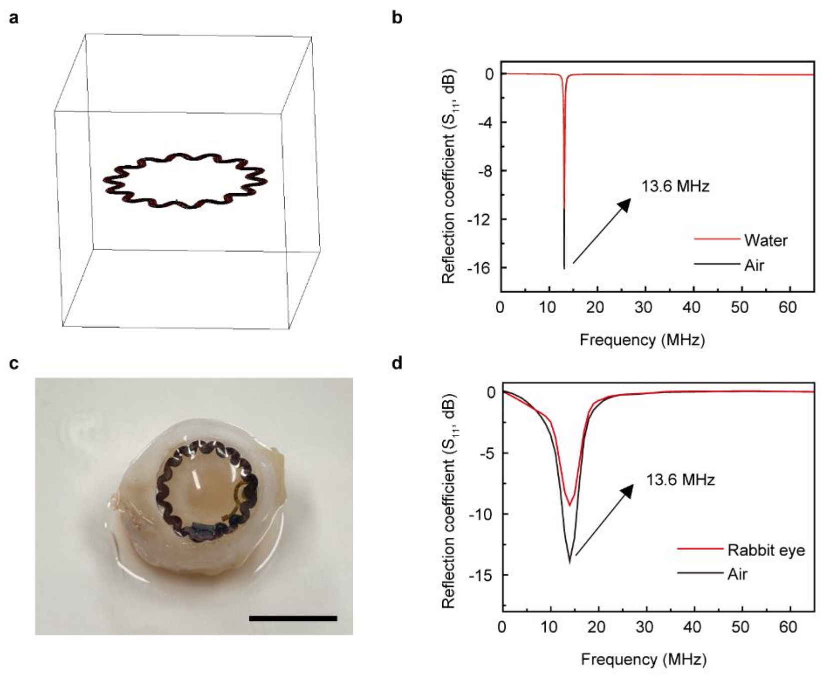

Figure S7. Electromagnetic characterizations of smart contact lenses. a) Illustrations of a serpentine antenna in HFSS simulation program. b) Resonance frequencies of the serpentine antenna resulted by the simulation according to the different medium (red, water; black, air). c) Photograph of the smart contact lens worn on a rabbit eye. Scale bar, 1 cm. d) Resonance frequencies of the smart contact lens in the air (black) and when worn on a rabbit eye (red).

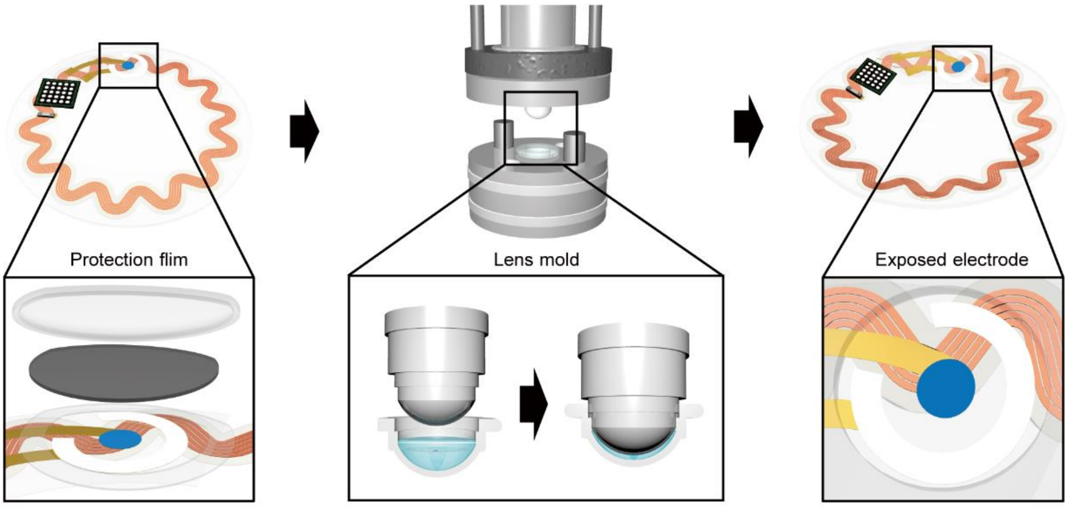

Figure S8. Schematic illustrations of the lens molding process with a biosensor protection film. A protection film was used to cover the cholesterol biosensor for immobilization of the enzyme after the contact lens molding process. Since the molding process includes thermal curing over 70˚C, the activity of the enzyme can be degraded if the immobilization proceeds before the molding process. The protection film was removed after transforming into the contact lens shape.

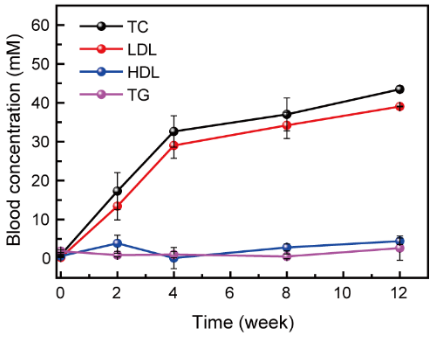

Figure S9. Changes in blood lipid profiles of cholesterol diet-fed rabbits. Total cholesterol (TC, black), low-density lipoprotein-cholesterol (LDL, red), high-density lipoprotein-cholesterol (HDL, blue) and triglyceride (TG, pink). The error bars represent the standard deviations (n = 4).

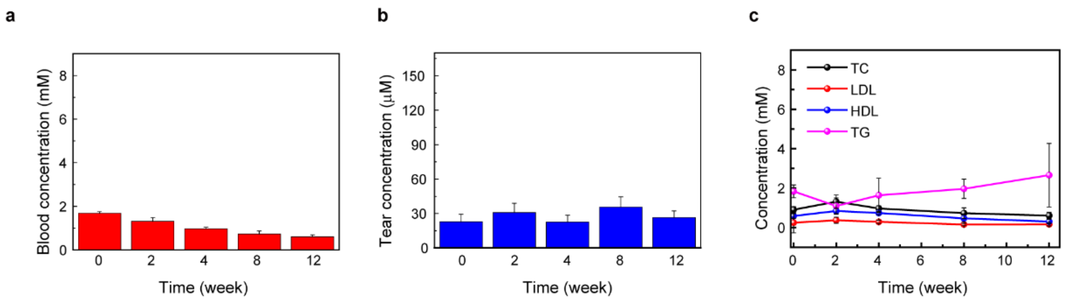

Figure S10. Cholesterol concentrations of the regular diet-fed rabbits. a) Variations of total cholesterol concentrations in blood of the regular diet-fed rabbits. b) Changes in free cholesterol concentrations of tear fluids of the regular-diet rabbits. c) Changes in blood lipid profiles of the regular diet-fed rabbits. Total cholesterol (TC, black), low-density lipoprotein-cholesterol (LDL, red), high-density lipoprotein-cholesterol (HDL, blue) and triglyceride (TG, pink). The error bars represent the standard deviations (n = 4).

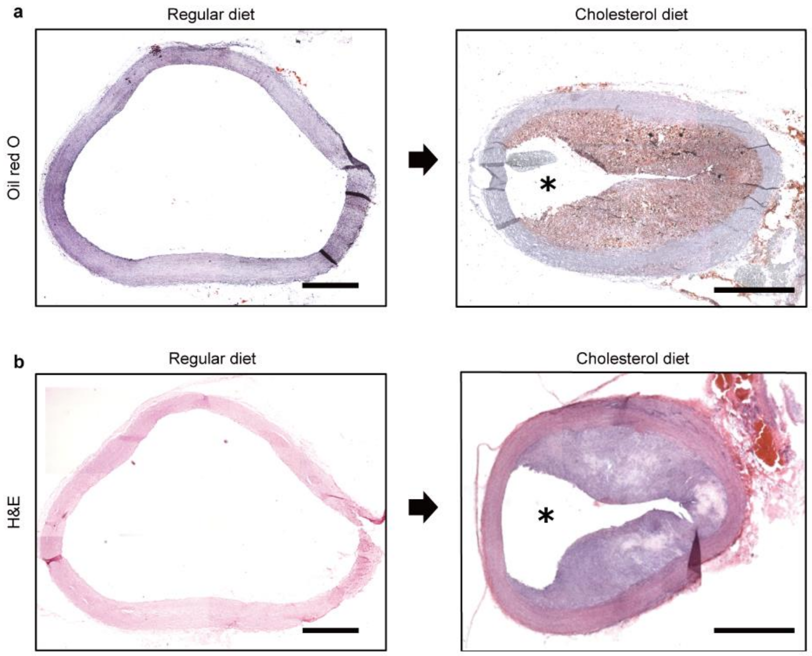

Figure S11. Aortic sections in regular and cholesterol diet-fed rabbits. a) Optical microscope images of aortic sections in regular (left) and cholesterol (right) diet-fed rabbits stained with oil red O staining, b) hematoxylin and eosin (H&E). The narrowed lumen (∗) of blood vessels due to plaque caused by cholesterol diet. Scale bars, 1mm.

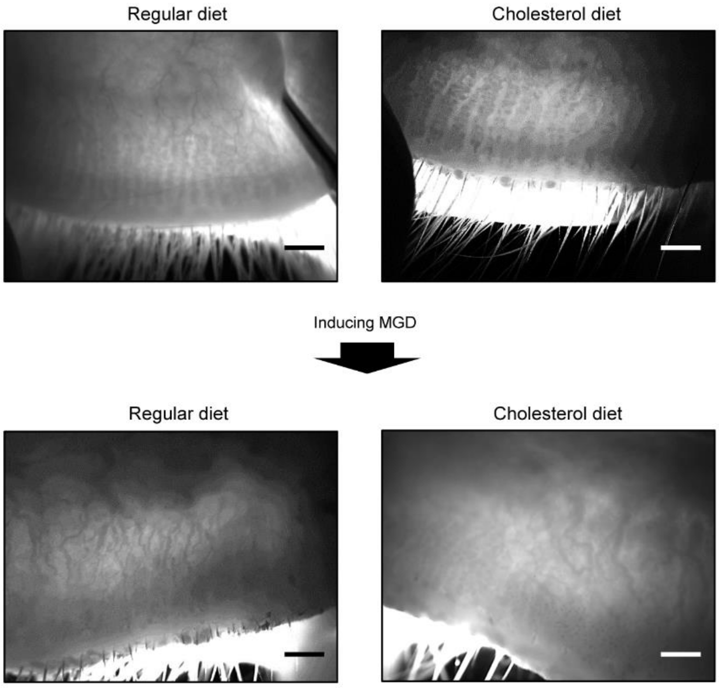

Figure S12. Meibography of regular (left) and cholesterol (right) diet-fed rabbits before/after inducing MGD.

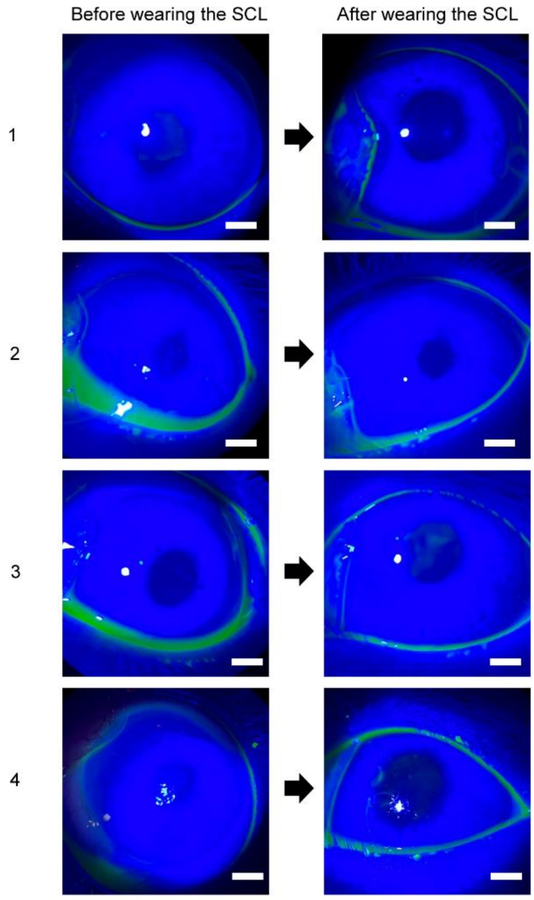

Figure S13. Immunofluorescence images of the cornea of rabbits before/after wearing the SCL. The immunofluorescence images of the cornea were acquired from four regular chow diet rabbits, and the numbers indicated each individual rabbit. Scale bars, 2 mm.

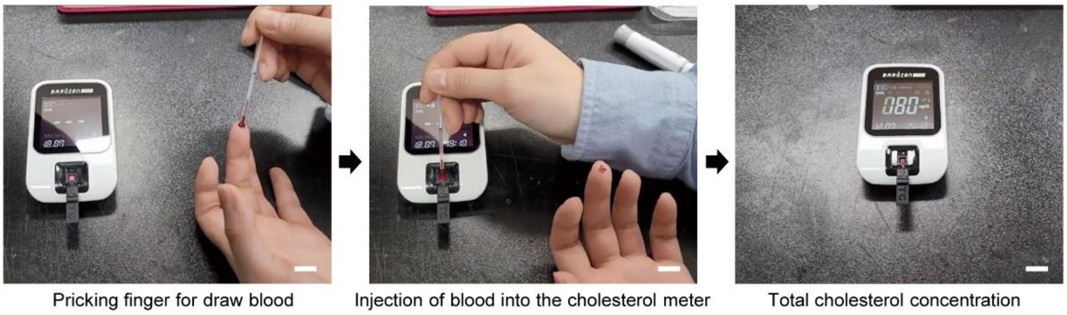

Figure S14. Photographs of the blood cholesterol measurement using a commercial cholesterol meter. Scale bars, 2 cm.

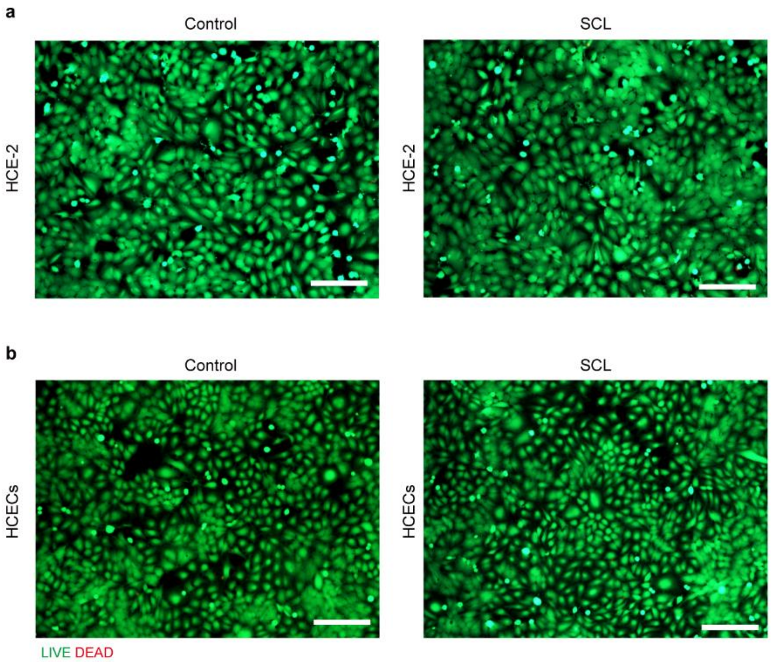

Figure S15. Fluorescence microscope images of human cells cultured in control and the SCL immersed medium, stained with calcein-AM. a) Human corneal cells (HCE-2). b) Human conjunctival cells (HCECs). Scale bars, 200 µm

Captions of Supporting Movie

Movie S1. *In-vivo* trial of the SCL for measuring free cholesterol. The SCL was worn on the human subject’s eye to measure wirelessly the concentration of free cholesterol in tear fluids.
